# Enhanced Patterned Cocatalyst TiO_2_/Fe_2_O_3_ Photoanodes for Water-Splitting

**DOI:** 10.1186/s11671-021-03529-8

**Published:** 2021-05-01

**Authors:** Wei-Hsuan Hung, Yung-Jen Teng, Chuan-Ming Tseng, Hien Thi Thai Nguyen

**Affiliations:** 1 Institute of Materials Science and Engineering, National Central University, No. 300 Jhong-da Rd., Jhongli City, 320 Taoyuan County Taiwan, ROC; 2 Department of Materials Science and Engineering, Feng Chia University, Taichung, 407 Taiwan, ROC; 3 Department of Materials Engineering, Ming Chi University of Technology, New Taipei City, 24301 Taiwan, ROC; 4 Center for Plasma and Thin Film Technologies, Ming Chi University of Technology, New Taipei City, 24301 Taiwan, ROC

**Keywords:** Bimetallic oxide, Periodic pattern, Hot-pressing process, Water splitting

## Abstract

In this study, we used a hot-pressing process to enhance the photocatalytic properties of TiO_2_/Fe_2_O_3_ bimetallic oxide with a periodic patterned structure on the surface to increase photon absorption for photocatalysis in the oxygen evolution reaction for water splitting. The hot-pressed samples show that combining the two metal oxides improves the absorption band edge of the electrode at different wavelengths. The patterned structure obtained using the hot-pressing process successfully improves photon absorption, resulting in a two-fold enhancement compared with a flat surface electrode.

**Supplementary Information:**

The online version contains supplementary material available at 10.1186/s11671-021-03529-8.

## Introduction

Photocatalytic decomposition for water splitting to produce oxygen is a widely studied light energy conversion system [1–4]. When photons of different wavelengths are irradiated onto a semiconductor photocatalyst, their energy agitates its valence band electrons, making them jump to the conduction band. A photo-generated hole is formed in the valence band, and the excited electrons in the conduction band undergo a reduction reaction with water molecules to produce hydrogen via the so-called hydrogen evolution reaction (HER) [5]. This hole dominates oxygen production via the so-called oxygen evolution reaction (OER) [6]. The edge of the conduction band of the semiconductor photocatalyst material must be above the H^+^/H_2_ reduction energy level. The photoelectrons in the photocatalyst can reduce water to hydrogen. However, because the oxidation–reduction potential difference of the water-splitting reaction is 1.23 eV, the valence band energy level of the photocatalyst must be below the oxidation energy level of O_2_/H_2_O to oxidize water to oxygen.

**Fig. 1:**
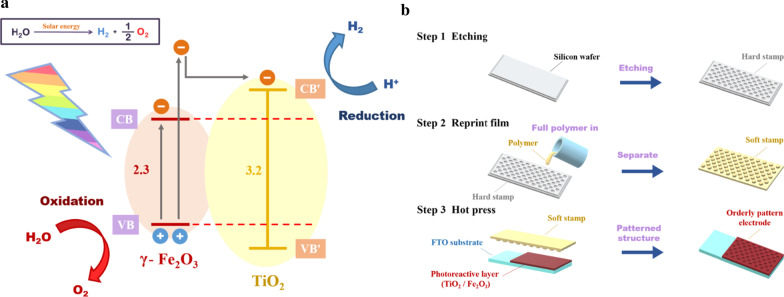
**a** Water-splitting reaction mechanism in TiO_2_/Fe_2_O_3_ bimetallic semiconductor system. **b** Fabrication of pattern using hot-pressing process

To achieve this goal, the adjustment of the required energy and the coordination of the solar radiation spectrum is important [1]. Most previous studies have used noble metals such as Pt and Au as catalysts [2, 5–7]; however, these are expensive and scarce, and therefore, studies have been conducted to find alternative catalytic materials. In this regard, typical semiconductor metal oxides have attracted much attention. Abundant metal oxides such as titanium dioxide (TiO_2_) [8, 9], WO_3_ [10, 11], BiVO_4_ [12, 13], CuO_2_ [14, 15], and ferric oxide (Fe_2_O_3_) [16, 17] enhance photon absorption through their n- or p-type semiconductor properties and energy gap matching; therefore, they show high photocatalytic efficiency over a large wavelength range. The photon energy of a specific wavelength can cause the separation of electron–hole pairs, further promoting the conversion of light energy into chemical energy. TiO_2_ [18–21] and Fe_2_O_3_ [22, 23] are commonly used for photocatalysis because they afford advantages such as simple preparation, high chemical stability, low cost, nontoxicity, and corrosion resistance; further, the energy gap of TiO_2_ (3.2 eV) shows good agreement with to the energy gap (2.2 eV) of Fe_2_O_3_ [24, 25], as shown in Fig. 1a. This property allows the bimetallic semiconductor formed by combining these two metal oxides to absorb more than 30% of the band gap effectively. Sunlight [26] can effectively enhance the photocatalytic effect of the electrode.

The dimensional structure of the electrode surface also influences the photoelectrochemical properties. In particular, periodic microstructures have attracted much interest in the field of optics. Yablonovitch and John described this concept in 1987 [27]. They aimed to design a medium that can capture photons to reduce energy consumption and waste. Through several years of research, they found that a medium with a particular periodic structure on the surface effectively traps photons [28, 29] without changing the intrinsic chemical properties of matter to obtain the required optical properties. Thus far, many studies on solar energy have selected materials with periodic structures to increase photon energy absorption [30]. Further, because a periodic microstructure drastically increases the reaction area of the electrode surface, the current response obtained will also be significantly improved.

In this study, we fabricated a simple pattern using a hot-pressing process onto the photoanode surface, as shown in Fig. 1b, and used an etching method to form an original substrate with a periodic surface structure. The original substrate is remolded by a polymer to serve as a new stamp substrate that is then used as a mold with the prepared layer of the TiO_2_/Fe_2_O_3_ cocatalyst. Finally, a hot-pressing process is performed to obtain a periodic microstructure. This process improves the carrier transfer rate through improved interface contact within the cocatalyst material and improves light absorption efficiency through additional light trapping and scattering from the surface patterns.

## Methods

### Preparation of the Fe_2_O_3_ and TiO_2_ Powder

FeCl_2_ and FeCl_3_ were dissolved in deionized water, stirred to form a solution, quickly poured into a sodium hydroxide solution, and finally stirred at 80 °C for 30 min. After the solution was thoroughly mixed, it was left to stand for 30 min until the product precipitated. The upper layer solution was removed; the precipitate was washed with acetone, ethanol, and deionized water; and it was dried at 120 °C for 12 h to obtain Fe_3_O_4_ (black powder). This powder was dissolved in alcohol and stirred vigorously for 30 min to obtain a reddish-brown Fe_2_O_3_ suspension solution. Finally, the precipitated Fe_2_O_3_ was placed in a quartz boat that in turn was placed in a sintering furnace at 450 °C for 3 h and then cooled to room temperature naturally to obtain Fe_2_O_3_ powder with a hematite phase. A TiO_2_ precursor solution was obtained by the addition of tetraethyl titanic acid to n-propanol to prepare a precursor solution followed by the addition of sulfuric acid and stirring at room temperature, allowing it stand to at 25 °C for 2 h to form a translucent gel, placing in an oven at 50 °C, reheating it, and naturally cooling it to room temperature.

### Preparation of the Bimetallic Oxide Colloidal Solution

Finally, we prepared 7 wt% polyvinyl alcohol (PVA), added 1 mL of deionized water, and placed it on a hot plate at 120 °C for 30 min. Then, we stirred the PVA to make it effectively dissolve in deionized water to obtain solution A. We prepared 20 mg of Fe_2_O_3_ powder and 98 µL of TiO_2_ solution to dissolve in 1 mL of N-methyl-2-pyrrolidone (NMP), placed it in an ultrasonic oscillator, shook it for 30 min to obtain fixed solutions, and placed them in an ultrasonic oscillator for 30 min to obtain the final semiconductor bimetallic oxide colloidal solution.

### Preparation of the Periodic Structure on the Electrodes

We used the imprint lithography process to fabricate the silicon wafer for the soft stamp [31–33]. Furthermore, to prepare the soft stamp, we first used acetone, ethanol, and water to vibrate the silicon wafer after the 20-min etching process to clean the board and then placed it on a heating plate at 40 °C for drying. Simultaneously, the epoxy resin was activated and then laid flat on the original substrate surface until it dried. After drying, the epoxy resin was torn off from the original substrate to obtain the required soft stamp. We applied 100 µL of the semiconductor bimetallic oxide colloidal solution to the TiO_2_ film surface and kept it at room temperature for 1 h until the colloidal solution changed to a jelly-like state, and then, we performed the hot-pressing process for 15 min. Finally, the patterned photoanode was placed in a sintering furnace at 500 °C for 3 h in an argon atmosphere to obtain the patterned photoanode with a periodic structure. The OER performance of the photoanode was examined using the three-electrode connection method. The system included the working electrodes, a counter electrode (carbon rod), and a reference electrode (Ag/AgCl) in 1 M KOH as the electrolyte.

## Results and Discussion

**Fig. 2:**
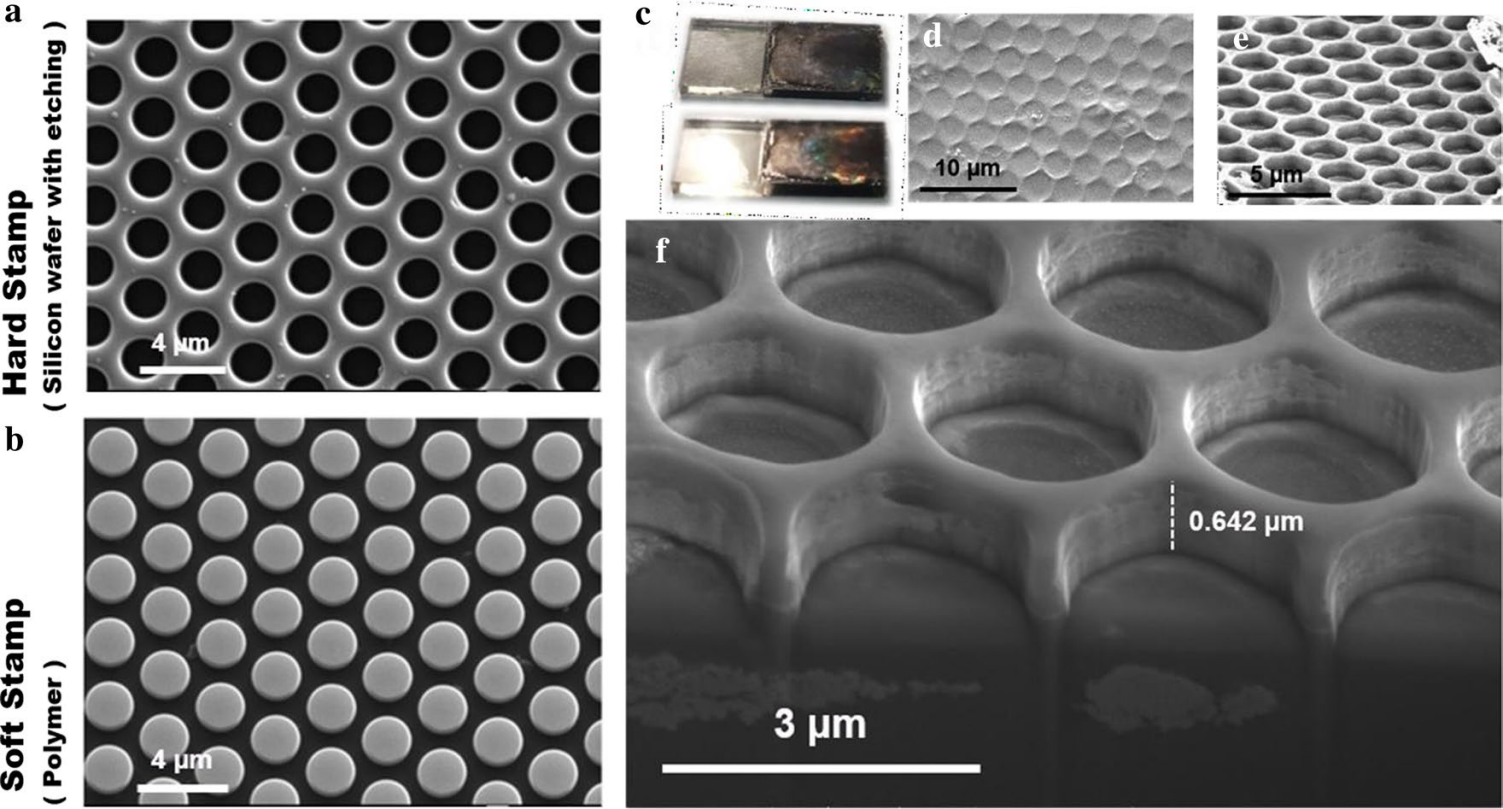
**a** SEM image of silicon wafer prepared using etching method. **b** Soft stamp made using silicon wafer with inverse pillar structure. **c** Photos captured with and without light irradiation. **d**–**e** SEM image under different magnifications and angles. **f** Cross-sectional image of electrode surface of TiO_2_/Fe_2_O_3_ ordered patterned photoanode

The surface-patterned structure was verified as shown in Fig. 2. Figure 2a shows a scanning electron microscope (SEM) image of the silicon wafer as a mother mold substrate. The surface had periodically arranged circular holes, each having an elongated 2-µm aperture. Figure 2b shows an image of the corresponding inverse pattern on the epoxy resin surface. The epoxy resin successfully replicated the whole structure from the original pattern of the Si substrate, which correspondingly showed periodically arranged cylindrical structures with a diameter of 2 µm. Finally, we examined whether the corresponding patterned periodic structure is transferred to the electrode surface via the hot-pressing process. Figure 2c shows the patterned TiO_2_/Fe_2_O_3_ photoanode before and after visible light irradiation. This figure shows that the electrode surface looks black when it is not illuminated. However, it shows a noticeable rainbow color under visible light irradiation, implying that the incident light is significantly trapped and refracted many times in the periodic patterned structure. Figure 2d, e presents SEM images of the surface of a patterned TiO_2_/Fe_2_O_3_ photoanode under different magnifications and angles. The photoelectrode surface exhibited a cycle similar to that of a silicon wafer motherboard. The pore size was approximately 2 µm, confirming that we successfully imprinted periodically patterned microstructures on the electrode surface. Finally, Fig. 2f presents a cross-sectional image produced by cutting the electrode surface using a focused ion beam (FIB). The cross-sectional image also shows the circular hole shape of this periodic patterned structure, with the hole depth being 0.642 µm. We also successfully used the anodic aluminum oxide as a stamp to fabricate a smaller pattern, and the SEM images can be found in Additional file 1: Fig. S1.

**Fig. 3:**
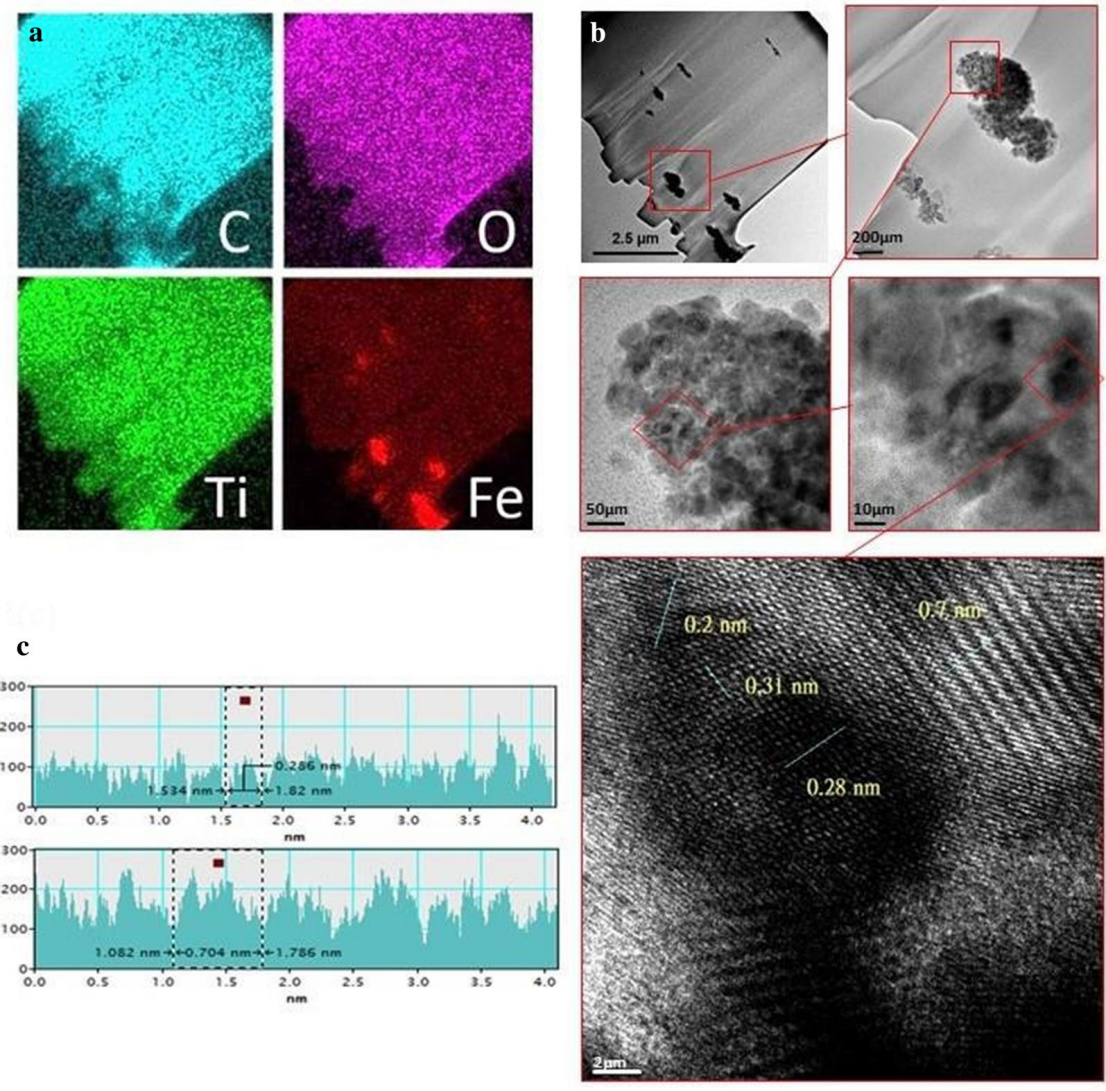
FIB-TEM image of TiO_2_/Fe_2_O_3_-ordered patterned photoanode with **a** EDS mapping of C, O, Ti, and Fe. **b** STEM images with different magnifications. **c** Analysis of Fe_2_O_3_ and TiO_2_ lattices

To characterize the proposed TiO_2_/Fe_2_O_3_ patterned photoanode, we conducted FIB-transmission electron microscope (TEM) analysis. Figure 3a presents the result of the element distribution analysis (EDS mapping) of the TiO_2_/Fe_2_O_3_ patterned photoanode. Fe, Ti, and O were uniformly distributed in the electrode, and the C signal arose from the PVA and NMP binders; however, this did not affect the distribution of the primary materials, namely TiO_2_ and Fe_2_O_3_. Figure 3b presents STEM images obtained under different magnifications. TiO_2_ and Fe_2_O_3_ powders exhibited granular morphologies. As shown in Fig. 3c, the lattice parameters of Fe_2_O_3_ and TiO_2_ were determined through the analysis to be 0.28 and 0.31 nm, respectively, indicating that the hot-pressing process created lattice distortion in both Fe_2_O_3_ and TiO_2_.

**Fig. 4:**
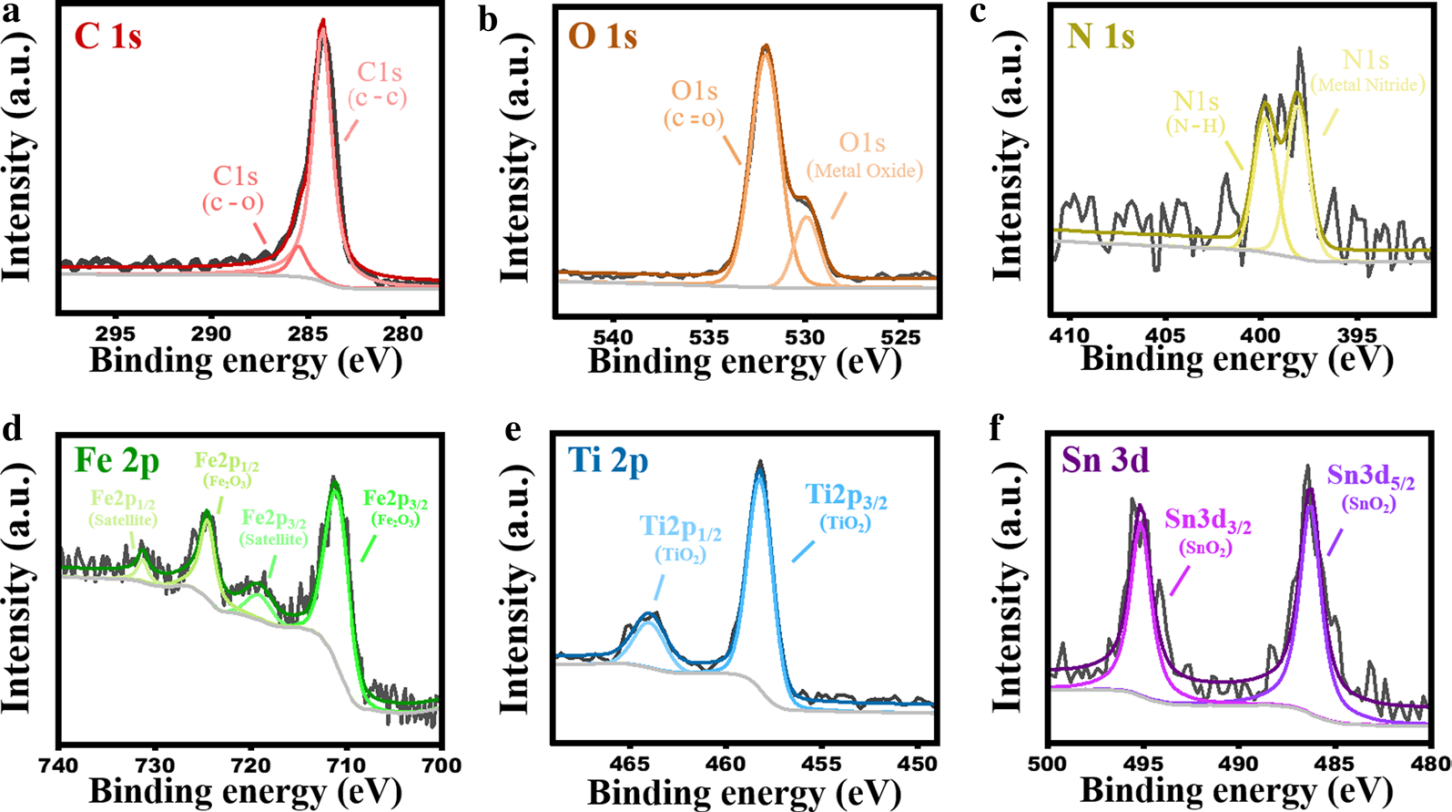
XPS spectra of TiO_2_/Fe_2_O_3_-ordered patterned photoanode for **a** C 1s, **b** O 1s, **c** N 1s, **d** Fe 2p, **e** Ti 2p, and **f** Sn 3d

Furthermore, we performed X-ray photoelectron spectroscopy (XPS) to determine the chemical states of elements. Figure 4 presents the results of the fine scan spectrum analysis performed using XPS for the six elements in the photoanode, and the full XPS survey spectrum can also obtain in Additional file 1: Fig. S2. In Fig. 4a, the C 1s orbital shows signals corresponding to a C–C single bond and a C–O single bond at a binding energy of 284.9 eV. In Fig. 4b, the O 1s orbital shows a signal of the C=O double bond at a binding energy of 532.5 eV, confirming that many oxidized carbons exist on the electrode surface and a signal of the O from the oxides at a binding energy of 530 eV. In Fig. 4c, the N 1s orbital shows signals of the N–H bond at binding energies of 397.2 and 400 eV. The bonding N and metal ion may result from the bond between N and a small amount of transition metal elements is also seen. In Fig. 4d, Fe 2p2/3 and Fe 2p1/3 signals are seen at binding energies of 711.3 and 724.8 eV, respectively, and satellite peaks of Fe 2p2/3 and Fe 2p1/3 are seen at binding energies of 720 and 731.3 eV, respectively; these are typical Fe_2_O_3_ configuration signals. In Fig. 4e, Ti 2p3/2 and Ti 2p1/2 signals are seen at binding energies of 457.9 and 464.3 eV, respectively; these are generated by TiO_2_. In Fig. 4f, Sn 3d3/2 and Sn 3d5/2 signals are seen at binding energies of 285.9 and 495.1 eV, respectively; these are generated by the SnO_2_ substrate.

**Fig. 5:**
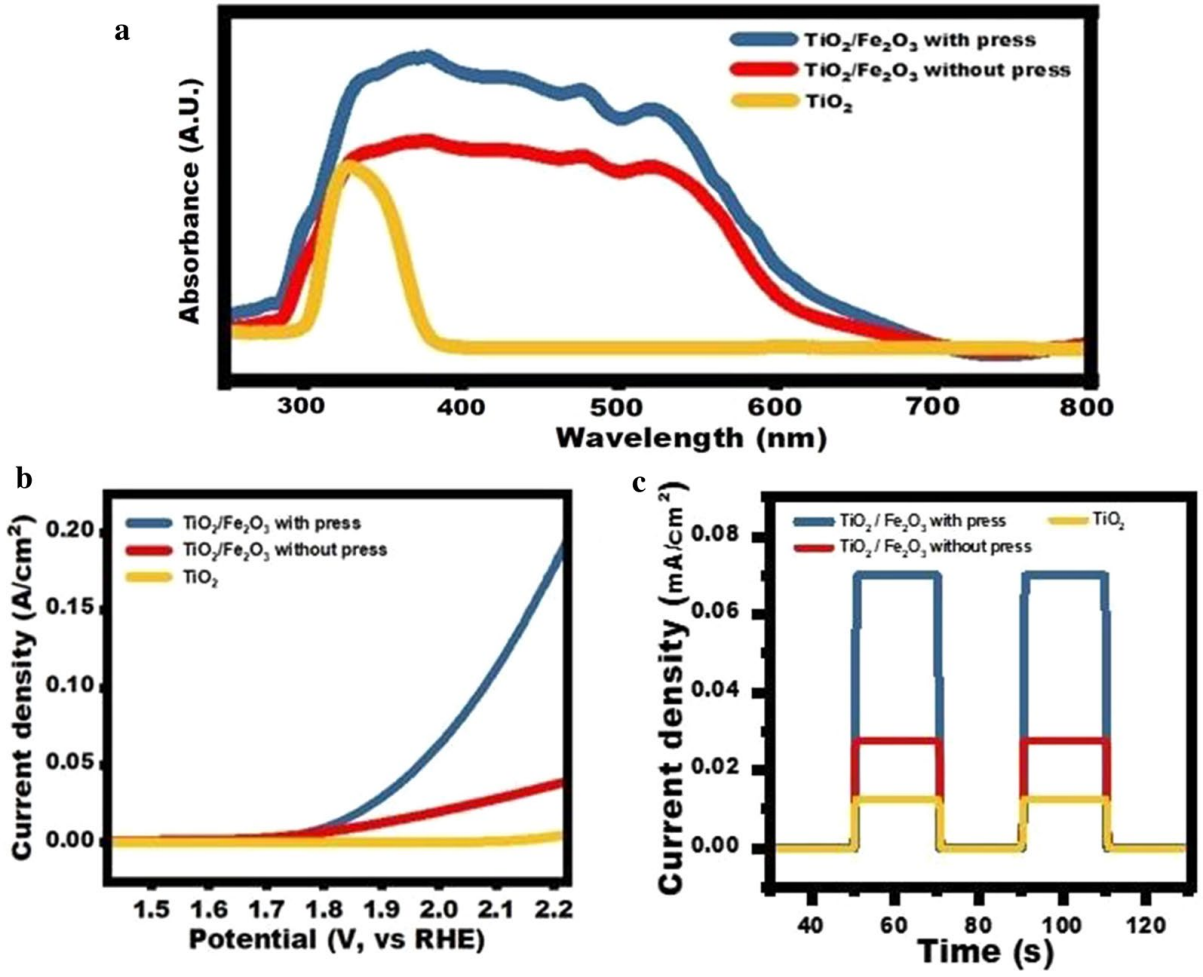
**a** UV–Vis absorption spectra. **b** LSV swipe scan. **c** Photoresponses of different photoanodes

To demonstrate the effect of patterned structures on the light absorption of the photoanode, we performed ultraviolet–visible spectroscopy (UV–Vis) before and after the hot-pressing process, as shown in Fig. 5a. Owing to the cocatalyst effect of the TiO_2_ and Fe_2_O_3_ metal oxides, the photoanode demonstrated light absorption over a broad range of 400–600 nm. Compared with the electrode before the patterning process, the photoanode exhibited additional light absorption owing to enhanced light scattering and absorption from the periodic patterned structure on the surface. This enhancement is also reflected in the linear scanning voltammetry (LSV) shown in Fig. 5b; the TiO_2_/Fe_2_O_3_ sample produced using the hot-pressing process exhibited the highest reaction current during the LSV scan. Furthermore, the EIS measurement and the Tafel slope can found in the Additional file 1: Figs. S3 and S5. Besides, we performed a photoresponse study under zero bias and white light irradiation, and this sample showed two-fold improvement compared with the TiO_2_/Fe_2_O_3_ sample produced without using the hot-pressing process and seven-fold current improvement compared with TiO_2_ only, as shown in Fig. 5c. We also selected the green laser with a wavelength of 532 nm and red laser with 633 nm for measurement, and the result can be found in Additional file 1: Fig. S4.

## Conclusion

In this study, we demonstrated a simple hot-pressing process to fabricate a periodic pattern on a TiO_2_/Fe_2_O_3_ cocatalyst bimetallic oxide photoanode. A clear periodic pattern of holes was reproduced on the photoanode surface. A broadband UV–Vis absorption spectrum of the TiO_2_/Fe_2_O_3_ bimetallic oxide was obtained, and it showed light absorption over a broad range of 400–600 nm. Finally, the TiO_2_/Fe_2_O_3_ cocatalyst with a patterned surface exhibited a significantly enhanced photocurrent owing to the additional light absorption and scattering from the surface structure.

## Supplementary Information

**Additional file 1.** **Figure S1.** SEM image and cross-sectional image of anodized aluminum template at different voltages (a) 30 V, (b) 60 V, (c) 90 V, (d) 120 V. And the corresponding transfer SEM images on the surface of epoxy resin, (a2) 30 V, (b2) 60 V, (c2) 90 V, (d2) 120 V. **Figure S2.** The full XPS survey spectrum analysis chart of patterned TiO_2_/Fe_2_O_3_ photoanode. **Figure S3.** The EIS measurement compares the difference between TiO_2_/Fe_2_O_3_ photoanode before and after hot pressing process. **Figure S4.** Compare the photocurrent response of patterned TiO_2_/Fe_2_O_3_ photoanode under two different laser irradiations. **Figure S5.** Tafel slope of the TiO_2_/Fe_2_O_3_ photoanodes with/without pattern.

## Data Availability

All data generated or analyzed during this study are included in this published article [and its supporting information files].

## Abbreviations

OER: Oxygen evolution reaction
HER: Hydrogen evolution reaction
PVA: Polyvinyl alcohol
NMP: N-methyl-2-pyrrolidone
SEM: Scanning electron microscope
FIB: Focused ion beam
TEM: Transmission electron microscope
EDS: Energy dispersive spectroscopy
STEM: Scanning transmission electron microscopy
XPS: X-ray photoelectron spectroscopy
UV–Vis: Ultraviolet-visible spectroscopy
LSV: Linear scanning voltammetry
