# Supplementary material for: Enhanced Patterned Cocatalyst TiO2/Fe2O3 Photoanodes for Water-Splitting
Source: Nanoscale Res Lett. 2021 May 1;16:76. doi: 10.1186/s11671-021-03529-8 (PMC8088417; doi:10.1186/s11671-021-03529-8)
Supplement: Supplementary file 1 — Additional file 1. Figure S1. SEM image and cross-sectional image of anodized aluminum template at different voltages (a) 30 V, (b) 60 V, (c) 90 V, (d) 120 V. And the corresponding transfer SEM images on the surface of epoxy resin, (a2) 30 V, (b2) 60 V, (c2) 90 V, (d2) 120 V. Figure S2. The full XPS survey spectrum analysis chart of patterned TiO2/Fe2O3 photoanode. Figure S3. The EIS measurement compares the difference between TiO2/Fe2O3 photoanode before and after hot pressing process. Figure S4. Compare the photocurrent response of patterned TiO2/Fe2O3 photoanode under two different laser irradiations. Figure S5. Tafel slope of the TiO2/Fe2O3 photoanodes with/without pattern. [file 11671_2021_3529_MOESM1_ESM.pdf]

Enhanced Patterned Cocatalyst TiO_2/Fe_2O_3 Photoanodes for Water-splitting

Wei-Hsuan Hung[1, *], Yung-Jen Teng[2], Chuan-Ming Tseng[3, 4, *], Thi Thai Hien Nguyen[1]

[1]Institute of Materials Science and Engineering, National Central University,
No. 300 Jhong-da Rd., Jhongli City 320, Taoyuan County, Taiwan, ROC.

[2] Department of Materials Science and Engineering, Feng Chia University,
Taichung 407, Taiwan, ROC.

[3] Department of Materials Engineering, Ming Chi University of Technology,
New Taipei City, 24301, Taiwan, ROC.

[4] Center for Plasma and Thin Film Technologies, Ming Chi University of Technology,
New TaipeiCity 24301, Taiwan

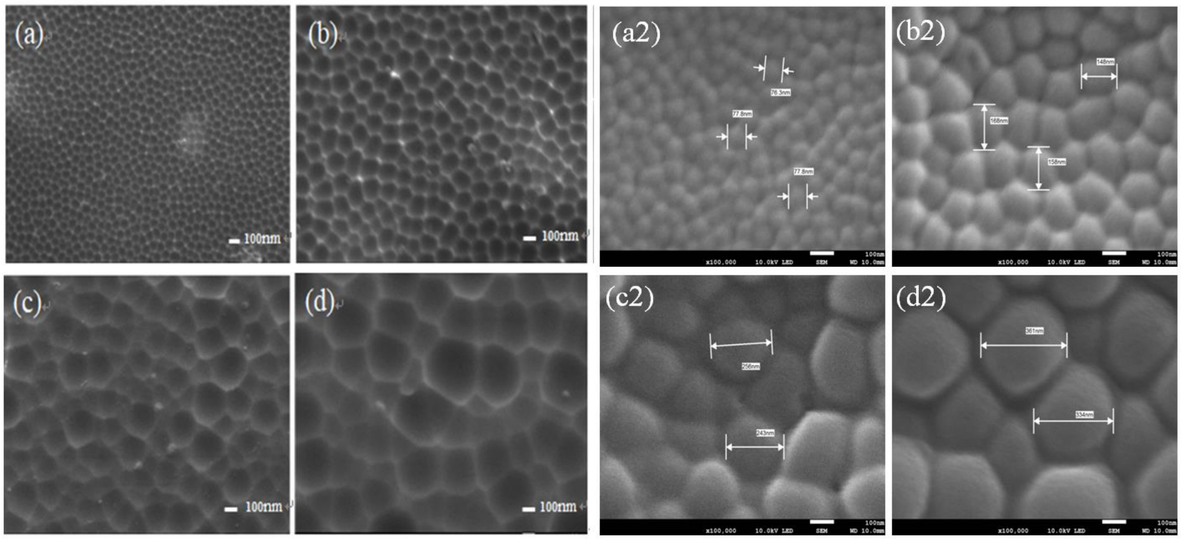

Figure S1. SEM image and cross-sectional image of anodized aluminum template at different voltages (a) 30 V, (b) 60 V, (c) 90 V, (d) 120 V. And the corresponding transfer SEM images on the surface of epoxy resin, (a2) 30 V, (b2) 60 V, (c2) 90 V, (d2) 120 V.

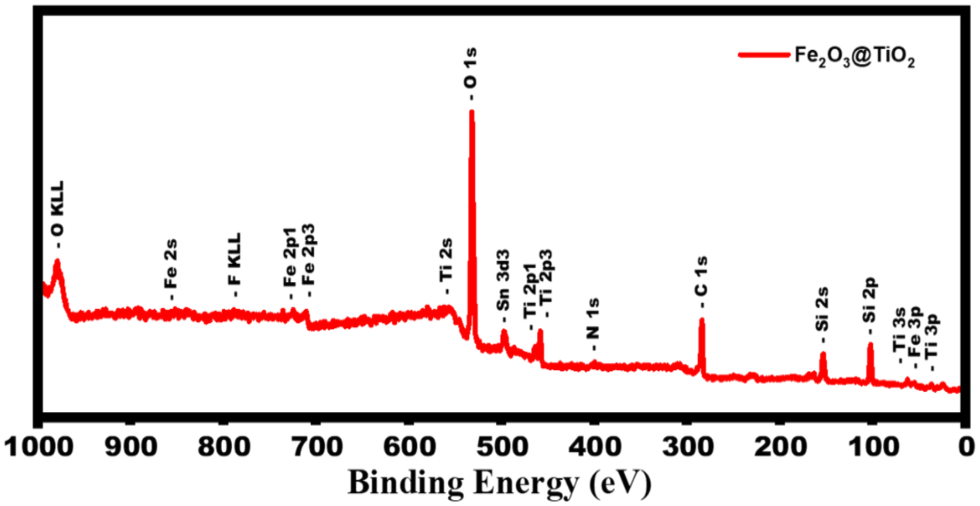

Figure S2. The full XPS survey spectrum analysis chart of patterned TiO_2/Fe_2O_3 photoanode.

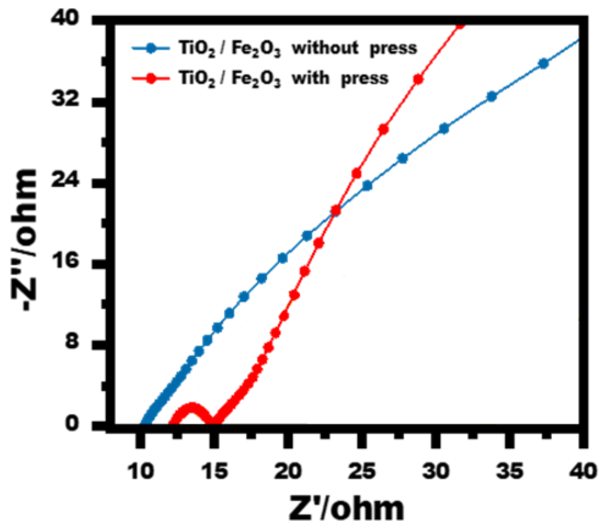

Figure S3. The EIS measurement compares the difference between TiO_2/Fe_2O_3 photoanode before and after hot pressing process.

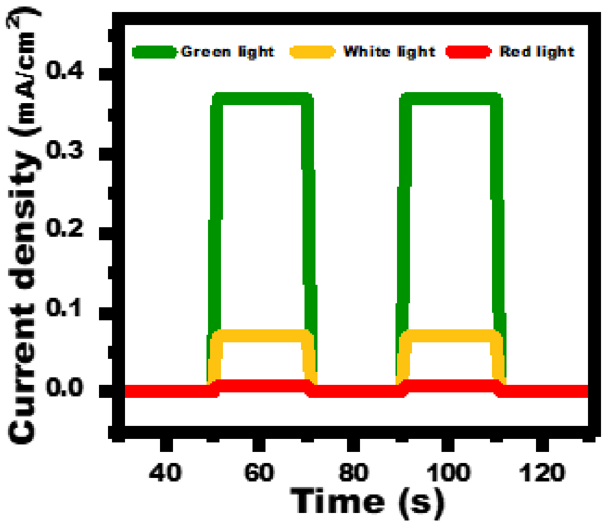

Figure S4. Compare the photocurrent response of patterned TiO_2/Fe_2O_3 photoanode under two different laser irradiations.

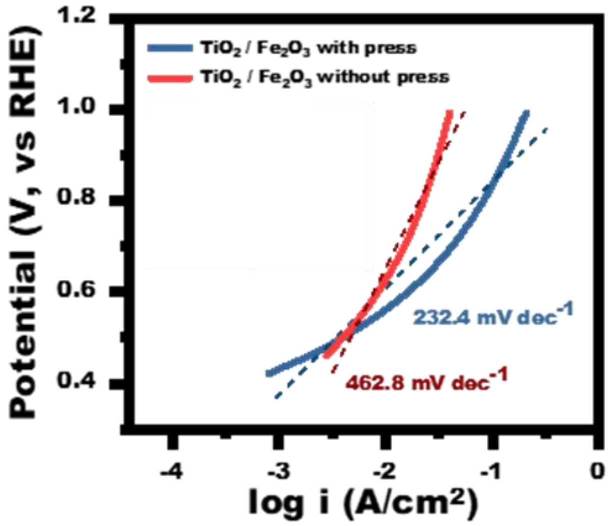

Figure S5. Tafel slope of the TiO_2/Fe_2O_3 photoanodes with/without pattern.
